# Colorectal carcinogenesis in the Lynch syndromes and familial adenomatous polyposis: trigger events and downstream consequences

**DOI:** 10.1186/s13053-025-00305-y

**Published:** 2025-01-23

**Authors:** Pål Møller, Aysel Ahadova, Matthias Kloor, Toni T. Seppälä, John Burn, Saskia Haupt, Finlay Macrae, Mev Dominguez-Valentin, Gabriela Möslein, Annika Lindblom, Lone sunde, Ingrid Winship, Gabriel Capella, Kevin Monahan, Daniel D. Buchanan, D. Gareth Evans, Eivind Hovig, Julian R. Sampson

**Affiliations:** 1 Department of Tumor Biology, Institute of Cancer Research, The Norwegian Radium Hospital Oslo University Hospital, 0379 Oslo, Norway; 2 Department of Applied Tumour Biology, Institute of Pathology, Heidelberg University Hospital, Heidelberg, Germany; 3 Faculty of Medicine and Health Technology, Tays Cancer Centre, Applied Tumor Genomics Research Program, Research Programs Unit, Tampere University, Tampere University Hospital, University of Helsinki, Helsinki, Finland; 4 Department of abdominal surgery, Helsinki University Hospital, Helsinki, Finland; 5 Faculty of Medical Sciences, Newcastle University, Newcastle Upon Tyne, NE1 7RU UK; 6 Colorectal Medicine and Genetics, The Royal Melbourne Hospital, Melbourne, Australia; 7 Department of Medicine, Melbourne University, Melbourne, Australia; 8 Surgical Center for Hereditary Tumors, University Witten-Herdecke, Ev. Bethesda Khs Duisburg, Herdecke, Germany; 9 Department of Molecular Medicine and Surgery, Karolinska Institutet, Stockholm, 171 76 Sweden; 10 Dept Clinical Genetics, Karolinska University Hospital, Stockholm, Sweden; 11 Department of Clinical Genetics and Clinical Cancer Research Center, Aalborg University Hospital, Aalborg, 9000 Denmark; 12 Department of Biomedicine, Aarhus University, Aarhus, DK-8000 Denmark; 13 Genomic Medicine, The Royal Melbourne Hospital, Melbourne, Australia; 14 Department of Medicine, University of Melbourne, Melbourne, Australia; 15 Hereditary Cancer Program, Institut Català d’Oncologia-IDIBELL, L; Hospitalet de Llobregat, Barcelona, 08908 Spain; 16 Centre for Familial Intestinal Caner, Lynch Syndrome & Family Cancer Clinic, St Mark’s Hospital, London, UK; 17 Colorectal Oncogenomics Group, Department of Clinical Pathology, The University of Melbourne, Parkville, Vic Australia; 18 Victorian Comprehensive Cancer Centre, University of Melbourne Centre for Cancer Research, Parkville, Vic Australia; 19 Genomic Medicine and Family Cancer Clinic, Royal Melbourne Hospital, Parkville, Vic Australia; 20 Manchester Centre for Genomic Medicine, Division of Evolution, Infection and Genomic Sciences, University of Manchester, Manchester University NHS Foundation Trust, Manchester, M13 9WL UK; 21 Division of Cancer and Genetics, Cardiff University School of Medicine, Heath Park, Cardiff, CF14 4XN UK

**Keywords:** Inherited, Cancer, Colorectal, Lynch syndromes, LS, MSI, MSH2, MSH6, PMS2, APC, FAP, Familial adenomatous polyposis, Carcinogenesis, Colonoscopy

## Abstract

Carcinogenesis encompasses processes that lead to increased mutation rates, enhanced cellular division (tumour growth), and invasive growth. Colorectal cancer (CRC) carcinogenesis in carriers of pathogenic *APC* (*path_APC*) and pathogenic mismatch repair gene (*path_MMR*) variants is initiated by a second hit affecting the corresponding wild-type allele. In *path_APC* carriers, second hits result in the development of multiple adenomas, with CRC typically emerging after an additional 20 years. In *path_MLH1* and *path_MSH2* carriers, second hits lead to the formation of microscopically detectable, microsatellite unstable (MSI) crypts, from which CRC develops in about half of carriers over their lifetime, often without progressing through a diagnosable adenoma stage. These divergent outcomes reflect the distinct functions of. the *APC* and MMR genes. In *path_MLH1* and *path_MSH2* carriers, a direct consequence of stochastic mutations may be the occurrence of invasive growth before tumour expansion, challenging the paradigm that an invasive cancer must always have an non-invasive precursor. In contrast to other *path_ MMR* carriers, *path_PMS2* carriers who receive colonoscopic surveillance exhibit minimal increase in CRC incidence. This is consistent with a hybrid model: the initial mutation may cause an adenoma, and the second hit in the wild-type *PMS2* allele may drive the adenoma towards become cancerous with MSI. Since all mutational events are stochastic, interventions aimed at preventing or curing cancer should ideally target the initial mutational events. Interventions focused on downstream events are external factors that influence which tumour clones survive Darwinian selection. In Lynch Syndrome, surveillance colonoscopy to remove adenomas may select for carcinogenetic pathways that bypass the adenoma stage.

## Introduction

Familial Adenomatous Polyposis (FAP) (OMIM **#** 175100 ) and inherited microsatellite-unstable (MSI) cancer, the four Lynch syndromes (LS) ( OMIM **#** 120435, **#** 609310, **#** 614350 and **#** 614337) [1], were initially identified clinically [2, 3] but, as predicted almost 150 years ago [4], inherited pathogenic variants were subsequently established as the underlying causes of these and other forms of inherited cancer predisposition. About 25,000 papers have been published on pathogenic variants of *APC* and the mismatch repair genes (*path_APC* and *path_MMR* [5] that cause FAP and LS, respectively, making it difficult for the human mind to hold and consider all available information for interpretation. Instead, we considered similarities and differences between FAP and LS using the basic method of starting with knowledge that is accepted universally and examining the degree to which this can explain what we have actually observed, before discussing some more recent observations [6].

### Tissue differentiation and conditional probabilities

Cells in the human body are controlled with respect to when and where they divide and their functional capabilities. One way of thinking about this is in terms of genes that are active or inactive in different cells during early embryonic life and that result in tissue differentiation. When a differentiated cell divides, the two daughter cells inherit the parental cell’s differentiation patterns, which may be determined by epigenetic inactivation [7]. Such changes may modify the probabilities for further events occuring in the daughter cells and may be subjected to analyses based on the conditional probability paradigm [8], which is reflected in the mathematical method used in object-oriented computer programming [9]. Here one starts with a general object (the multipotent cell), which may gain attributes (loss or addition of capabilities) providing sub-entities (clones) with these different capabilities, with further clones with new attributes emerging subsequently, and so on. Notably, this paradigm includes the expectation that all steps in the carcinogenetic process will be represented within a final cancer. This paradigm has, however, been challenged, for example by a recent report that *SOX17*, that is normally active during tissue differentiation in early embryonic life, may be reactivated early in the carcinogenetic process, conferring resistance to immune surveillance, but may not be transcribed later [10].

### Carcinogenesis, complex rearrangements and selection

Carcinogenesis is a multifactorial process [11–13] one aspect of which is the occurrence of mutations creating variants that are transmitted to successive generations of cells and that may modify the probabilities that other mutations will occur later. This paradigm may be used as the basis for mathematical modelling to better understand carcinogenesis [14]. “Driver mutation” is a term used to describe changes in the sequences of genes that cause cells to become cancer cells and grow and spread in the body [15]. Their effects include three different classes of events that permit escape from the normal order of cells and tissues: (1) an event (mutation) that accelerates the accumulation of subsequent mutations needed to change the properties of cells and that we refer to below as “*increased mutation rate”*, (2) an event (mutation) causing the cells to divide more and/or die less “increased cell number” (3) an event (mutation) causing cells to move from and survive outside their normal space (“*invasive growth”*). If a mutated cell does not divide, it does not cause a tumour, but it may survive without dividing and later aquire further mutations causing uncontrolled cell division. Alternatively, mutations may cause a cell to die. Each of the three classes of carcinogenetic event may be caused by different mutations in many different genes. A cancer may be considered as a population of cells with sub-populations resulting from complex genetic drift and selection, as is recognized in the field of population genetics [16, 17]. However, during carcinogenesis, complex rearrangements may occur and Darwinian selection may remove intermediate carcinogenetic steps such that they may not be present in the final cancer [18–21]. An example of a complex event is chromothripsis-mediated loss of *MLH1* [22].

### Both FAP and LS cancers predominantly occur in cells derived from embryonic endodermal epithelium

Why *path_APC* variants cause cancer predominantly in the colorectal epithelium, and *path_MMR* variants in this and additional organs derived from embryonic endodermal cells is not known. However, it seems likely that early embryonic organogenesis incapacitates alternative mechanisms in these tissues that might otherwise compensate for lost APC or MMR functions [1]. The epigenetic silencing of genes during organogenesis may be considered as the somatic acquisition of attributes that are transmitted through mitosis and change gene functions, just as mutations do. A recent report indicates that epigenetic changes that silence *SOX17* following early tissue differentiation may be reversed and the gene transcribed at different stages of carcinogenesis [10].

### FAP, LS and the adenoma-carcinoma paradigm

The *APC* gene encodes a multidomain protein that plays roles in tumour suppression, including regulation of Wnt signalling and beta-catenin activity (OMIM # *611431). A linear progression to CRC involving the three classes of carcinogenic events noted above has been demonstrated in *path_APC* carriers. Initially, a second-hit affects the wild-type *APC* allele. Its nature depends on that of the inherited mutation, such that beta-catenin binding is reduced but not abolished (the “just right” signalling hypothesis) [23]. This causes the cell division rate to increase and, together with other changes including effects on the migration of cells up crypts and their loss to the gut lumen, leads to the development of an adenoma. The many cell divisions involved increases the cumulative number of mutations, and over time (typically 20 years or more) a very small proportion of adenomas will acquire mutations permitting invasive growth and become cancers. Exactly when cancer arises is the result of stochastic probabilities and cannot be foreseen at the individual level. CRCs originating this way usually exhibit chromosomal instability (CIN), a characteristic to which disturbed APC function appears to contribute [24, 25]. As adenomas in *path_APC* carriers are typically too numerous to be removed at colonoscopy, colectomy is advocated to prevent CRC [26].

At first, it was assumed that most CRCs in LS would also develop from adenomas (although adenomas are not usually a prominent feature in LS and hence the previous term Hereditary Non-Polyposis Colorectal Cancer, HNPCC). It was reasoned that the removal of adenomas through colonoscopy would prevent CRC in *path_MMR* carriers. However, it became evident some ten years ago that CRCs were still being diagnosed in *path_MMR* carriers, despite regular colonoscopy and removal of adenomas. The CRCs originating this way are usually MSI and not CIN. Assuming that adenomas in *path_MMR* carriers might spend only a short time in the adenoma stage (the accelerated adenoma-carcinoma paradigm in LS) [27], reduced intervals between colonoscopies were advocated [28]. But CRCs continued to occur.

Based on these observations, the Prospective Lynch Syndrome Database (PLSD, www.plsd.eu) was designed to record the prospective incidences of CRC (and other cancers) in path_MMR carriers receiving colonoscopy surveillance with removal of adenomas. Comparing the CRC incidences in PLSD with those determined by retrospective segregation analyses that included carriers who were not subjected to colonoscopy showed that CRC incidence was not reduced by colonoscopy with polypectomy in *path_MLH1* or *path_MSH2* carriers [29]. This conclusion was supported recently in a report on CRC incidences in *path_MMR* carriers ascertained through sequencing of constitutional DNA samples in the UK Biobank [30].

Analyses in PLSD and other series also showed that shorter intervals between colonoscopies did not reduce CRC incidence in *path_MLH1*, *path_MSH2* and *path_MSH6* carriers [31, 32]. On the other hand, very few CRCs were observed in the *path_PMS2* carriers followed in PLSD, suggesting that most CRCs in *path_PMS2* carriers might orginate in adenomas that could be identified and removed at colonoscopy [21].

### MSI cancers and dMMR/MSI crypts in *path_MMR* carriers

In parallel with these epidemiological studies, biological studies demonstrated that, as a consequence of second hit mutations affecting the wild-type *MMR* allele, *path_MLH1* and *path_MSH2* carriers may accumulate up to thousands of mismatch repair deficient (dMMR)/MSI colonic crypts with increasing age [14, 33]. In these carriers, MSI CRCs may develop from one or more of the dMMR crypts. In contrast to the second hits in *path_APC* carriers that cause increased cell division rates and adenomas, the second hits in the *path_MMR* carriers cause an increased mutation rate. It was assumed that mutations in genes controlling cell division rates would sometimes follow, leading to adenomas, and then in genes governing invasion, consistent with the accelerated adenoma-carcinoma paradigm. However, the sequence of events that lead to increased cell division and to invasion is debated [21]. Ahadova et al. [34] noted that in colonic crypt cells of *path_MLH1* carriers that already harbour a single *CTNNB1* mutation, copy number-neutral loss of heterozygosity encompassing a 5 Mb region of chromosome 3 that contains both the *MLH1* and *CTNNB1* genes leads to a “double second-hit” resulting in both dMMR/MSI and carcinogenic beta-catenin status, consistent with the earlier observation that *CTNNB1* mutations are much more common in *MLH1*- than *MSH2*-associated CRCs [35].

Carcinogenesis in *path_PMS2* carriers may be quite different. Their CRC risks are only slightly elevated [36] and their CRCs may develop from adenomas, enabling CRC prevention by colonscopic surveillance [21].

### Similarities and differences between FAP and LS

The different colorectal phenotypes in FAP and the LSs may reflect the different consequences of somatic second hit mutations in the wild-type *APC* and *MMR* genes. Not only do they result primarily in proliferation and increased mutation rates, respectively, both of which increase the probability for further mutations, but also the types of downstream mutations are distinct, reflecting the targeting of different DNA sequences and structures and CIN vs. MSI as the major forms of genomic instability. Carcinogenesis in both conditions involves events (the acquisistion of mutations) that confer the combination of increased mutation rate, increased proliferation and invasive growth, while the order of these events may differ.

A short time, or perhaps no time, in the macroscopic adenoma stage is likely to underly the failure of colonoscopic surveillance in CRC prevention in LS. Nonetheless, this intervention remains the mainstay of recommendations for clinical management [37], because the prognosis of CRC is good in *path_MMR* carriers who are subjected to regular colonoscopy with polypectomy [38, 39]. The risk of CRC in FAP appears higher than in LS and its prognosis less good [26, 38]. A major difference between FAP and LS is the immune response that is triggered by neoantigens in the MSI setting. The response may not only destroy dMMR crypts, but also MSI CRCs and its manipulation offers opportunities for cancer treatment and prevention in LS that are curently unavailable in FAP [1, 40, 41].

### Why focus on the triggers and early events in carcinogenesis?

Identifying and counteracting the triggers of carcinogenesis may contribute to strategies for cancer prevention and treatment. An example of preventing the trigger event is vaccination against HPV-associated cancers; an example of improving treatment by targeting the trigger event is the use of imatinib to inhibit the BCR-ABL fusion protein in chronic myeloid leukemia. There is no approach immediately at hand to prevent occurrence of the second-hit mutations that trigger carcinogenesis in FAP and LS but strategies to counter their consequences are being explored. These include vaccination against recurring neoantigens that characterise MSI cells as an approach to cancer prevention in LS [41]. By comparison with targeting early events in carcinogenesis, treatments targeting events further downstream may be inefficient due to positive selection for resistant clones arising through stochastic mutation.

### Chemoprevention

Acetylsalicylic acid has been demonstrated to reduce adenoma load in *path_APC* carriers [42] and to reduce CRC incidence in *path_MMR* carriers [43], but the mechanisms involved remain uncertain [44].

### Additional remarks on FAP and inherited MSI carcinogenesis

Endoscopy and surgery that are undertaken in *path_A*PC and *path_MMR* carriers as part of clinical care have facilitated the study of FAP and LS colorectal adenomas and revealed acquired mutations in diverse genes, including established cancer drivers [45, 46]. However, more informative insights into the nature and order of the earliest events in colorectal carcinogenesis in individuals with inherited colorectal cancer predispostion are starting to emerge from next generation sequencing studies of single colonic crypts [47–49]. In addition to demonstrating mutational signatures consistent with dMMR, a study in LS patients revealed mutations in *APC*, *KRAS* and other established cancer drivers in 20% of the crypts or glands from morphologically normal gut epithelium and a single morphologically normal crypt with dMMR.Further similar studies are required to provide a comprehensive picture of early colorectal carcinogenesis in FAP and LS.

## Conclusions

While the early driver events in both FAP and MSI cancers are second-hit mutations affecting their respective wild-type alleles, the consequences are different because of the different functions that are lost after the second-hits. In addition to the widely accepted paradigm of a stepwise process from adenomas to carcinomas in colonic epithelial tissue, we discuss information indicating that the carcinogenetic processes are stochastic and in LS, may not always include a precancerous adenoma stage. A consequence of complex mutational events and selection determining which clones survive may lead to the absence of some intermediate carcinogenetic steps in CRCs. Not all steps that lead to CRC may be understood by examining only the resultant CRCs. In inherited MSI cancers, the host immune system contributes to the selection of surviving clones that become CRCs. Colonoscopy may abort pathways to cancer with polypoid precursors, selecting for non-polypoid carcinogenetic pathways. Interventions to prevent or cure CRC should preferably target early events, because interventions targeting later events may be circumvented by alternative carcinogenetic processes.

## Data Availability

No datasets were generated or analysed during the current study.
